# Diffuse myocardial fibrosis in early forms of dilated cardiomyopathy: insights from T1 mapping cardiovascular magnetic resonance

**DOI:** 10.1186/1532-429X-15-S1-P147

**Published:** 2013-01-30

**Authors:** Fabian aus dem Siepen, Sebastian A Seitz, Mohamed A Abdelrazek, Evangelos Giannitsis, Hugo A Katus, Henning Steen, Hassan Abdel-Aty

**Affiliations:** 1 Universitatsklinikum Heidelberg, Heidelberg, Germany; 2 Radiology, Cairo university, Faculty of Medicine, Cairo, Egypt

## Background

The underlying myocardial injury in early forms of dilated cardiomyopathy (DCM) is unclear. Cardiovascular magnetic resonance (CMR) myocardial T1-mapping monitors the enlargement of the cardiac extra-cellular space and thus offers a non-invasive tool of quantitative measurement of myocardial fibrosis. We used T1-mapping techniques to explore the hypothesis that diffuse myocardial fibrosis is related to DCM.

## Methods

We investigated 77 DCM patients (55 ± years, 51 males) and 46 healthy controls (52 ± years, 23 males). Significant coronary artery disease was excluded invasively. All CMR examinations were performed using 1.5 T CMR scanner (Philips Achieva). Short axis slices covering the LV were acquired using SSFP sequence to measure volumes and ejection fraction. T1-relaxation times were measured before and 15 minutes after Gd-DTPA (Magnograf) injection (0.2 mmol/kg). T1-maps were created out of 11 mid-ventricular short axis views with increasing inversion times (TI;100 - 4400 msec.) using a single breath-hold modified Look Locker inversion recovery sequence (MOLLI, TR/TE=3.5/1.8 msec, flip angle = 35°) in late diastole. Early DCM with mild reduction of LVEF was defined as EF between 45-55% while severely depressed LVEF was defined as EF<30%. Delta-T1 was calculated as non-contrast myocardial T1-post-contrast myocardial T1.

## Results

**Figure 1:**
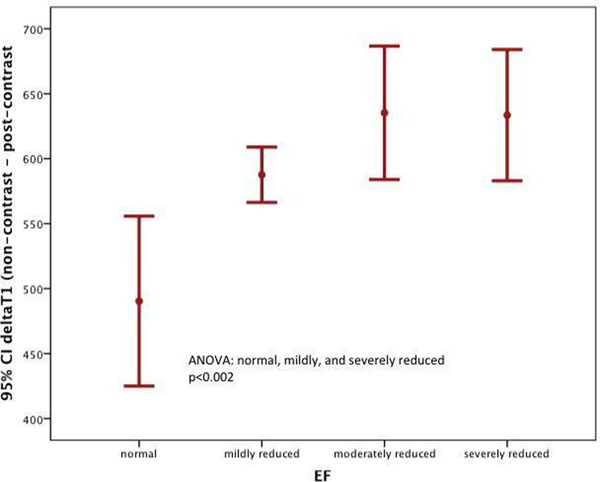


Mean LVEF was 43 ± 12% in DCM and 62 ± 4 in controls. Compared to controls, deltaT1 was significantly higher in 42 early DCM patients (588 ± 60 ms; vs. 490 ± 220ms; p=0.006). Patients with advanced DCM (n=10) had the highest delta-T1-values (634 ± 71 ms). Non-contrast-T1 (371 ± 164 ms vs. 399 ± 41 ms; p =ns) and post-contrast-T1 (1018 ± 40 ms vs. 1037 ± 45 msp=ns) were not significantly different between controls and patients with advanced DCM. ANOVA analysis showed significant differences between the groups (p<0.002, Figure 1). There was no significant association between the presence of late gadolinium enhancement (LGE) and the severity of LV dysfunction (31% of the patients in early DCM and 45% in severely depressed EF group; p=0.5). No significant differences in T1-values (non-contrast-, post-contrast- or delta-T1) between moderately and severely depressed EF were found (p=0.9).

## Conclusions

Diffuse myocardial fibrosis commences already at an early disease stage of DCM when LVEF is only mildly reduced. In contrast to focal fibrosis seen in LGE, diffuse fibrosis is more linked to the severity of LV dysfunction. Importantly, the differences between T1-values before and after contrast administration (delta-T1) appear to be a more reliable parameter in the assessment of myocardial fibrosis than a single time-point measurement.

## Funding

none

